# pSSAlib: The partial-propensity stochastic chemical network simulator

**DOI:** 10.1371/journal.pcbi.1005865

**Published:** 2017-12-04

**Authors:** Oleksandr Ostrenko, Pietro Incardona, Rajesh Ramaswamy, Lutz Brusch, Ivo F. Sbalzarini

**Affiliations:** 1 Center for Information Services and High Performance Computing, TU Dresden, Dresden, Germany; 2 Center for Advancing Electronics Dresden, TU Dresden, Dresden, Germany; 3 Chair of Scientific Computing for Systems Biology, Faculty of Computer Science, TU Dresden, Dresden, Germany; 4 MOSAIC Group, Center for Systems Biology Dresden, Dresden, Germany; 5 Max Planck Institute of Molecular Cell Biology and Genetics, Dresden, Germany; 6 Max Planck Institute for the Physics of Complex Systems, Dresden, Germany; Hebrew University of Jerusalem, ISRAEL

## Abstract

Chemical reaction networks are ubiquitous in biology, and their dynamics is fundamentally stochastic. Here, we present the software library pSSAlib, which provides a complete and concise implementation of the most efficient partial-propensity methods for simulating exact stochastic chemical kinetics. pSSAlib can import models encoded in Systems Biology Markup Language, supports time delays in chemical reactions, and stochastic spatiotemporal reaction-diffusion systems. It also provides tools for statistical analysis of simulation results and supports multiple output formats. It has previously been used for studies of biochemical reaction pathways and to benchmark other stochastic simulation methods. Here, we describe pSSAlib in detail and apply it to a new model of the endocytic pathway in eukaryotic cells, leading to the discovery of a stochastic counterpart of the cut-out switch motif underlying early-to-late endosome conversion. pSSAlib is provided as a stand-alone command-line tool and as a developer API. We also provide a plug-in for the SBMLToolbox. The open-source code and pre-packaged installers are freely available from http://mosaic.mpi-cbg.de.

This is a *PLOS Computational Biology* Software paper.

## Introduction

Modeling chemical reaction networks is key to unraveling the mechanisms underlying cell physiology and pathology [1–3]. Given the complexity of biochemical reaction networks and the large number of interacting species, computational studies are often necessary to guide experimental assays and to identify candidates for wet-lab perturbation. Numerical computer simulations provide full control over all parameters of the model, enabling “what if” studies that would be hard to address otherwise.

Such computational studies, however, are often complicated by the fact that molecules move and react stochastically. Biochemical reaction networks in sub-cellular compartments are frequently characterized by low copy numbers for some of the involved molecules, which leads to stochastic kinetics also of macroscopic quantities that can be qualitatively different from the deterministic predictions of standard reaction-rate equations [4–7]. In addition, biochemical reactions often incur a time delay (e.g., chaperoning through the nuclear pore complex, multistage binding, and mRNA translation), causing additional fluctuations in the molecular concentrations. Lastly, the spatial distribution of chemical species may play an important role within the same compartment, as is for example the case for diffusion in the endoplasmic reticulum [8]. A constructive role of fluctuations may also lie at the core of biological information processing, e.g., in transcription and translation [9, 10].

Accounting for the exact stochastic kinetics of a reaction network is typically done using exact stochastic simulation algorithms (SSAs). They sample species populations from the exact solution of the chemical master equation (CME) [11], preserving all information about the molecular fluctuations in the system, and their dynamics. To accommodate time delays, some of these algorithms have been extended accordingly [12]. Finally, a classical way of accommodating spatial heterogeneity is to subdivide the system into a number of well-mixed sub-spaces and to perform the reaction sampling in each of them according to the original scheme, augmented by jump reactions between sub-spaces in order to model diffusion. This classical algorithm is known as the next subvolume method (NSM) [13]. Together, these approaches provide a toolbox to account for stochastic effects in chemical reaction networks with elementary rates.

The use of this toolbox for simulating large reaction networks with sufficient statistics is mainly limited by the computational cost of the algorithms. Therefore, numerous improvements to the computational efficiency of exact SSAs have been presented in the past, notably the next reaction method (NRM) by Gibson and Bruck [14] and the SSA with Composition-Rejection Sampling (SSA-CR) [15]. While all other known variants of exact SSA have a computational cost that scales linearly with the number of reactions in the network [16], NRM reduced the runtime to scale with the logarithm of the number of reactions and SSA-CR further reduced it to constant-time. These improvements, however, only hold for weakly coupled networks [16]. For strongly coupled networks, the scaling of the computational cost remains linear with the number of reactions, which is unfavorable when simulating strongly coupled biochemical networks that contain many more reactions than species. A new class of SSAs based on factored-out reaction propensities has therefore been proposed [16] to improve the cost also for strongly coupled networks (see Partial-propensity methods in Sec. Design and implementation for the concept underlying this new class of methods). These so-called partial-propensity SSAs have a computational cost that scales at most linearly with the number of species, but not with the number of reactions, even for strongly coupled reaction networks. Two such algorithms are the partial-propensity direct method (PDM) [16] and the sorting partial-propensity direct method (SPDM) [16]. For weakly coupled networks, the computational cost is further reduced to constant-time using the partial-propensity SSA with Composition-Rejection Sampling (PSSA-CR) [17]. Partial-propensity methods [18] have also been extended to reactions with time delays (dPDM, the delay partial-propensity direct method) [19], and to spatiotemporal reaction-diffusion systems (PSRD, the partial-propensity stochastic reaction-diffusion method) [20]. Due to the state-of-the-art computational performance of partial-propensity methods, they are frequently used to benchmark newer SSAs [21–24]. In addition, particular instances of partial-propensity methods have been included in third-party software libraries, such as *ngss* [21] and *ssapredict* [24]. However, no complete and concise software implementation of all partial-propensity algorithms is available with support for delayed chemical reactions and spatiotemporal dynamics.

Here, we present the partial-propensity stochastic simulation algorithms library (pSSAlib), a fast, flexible, and user-friendly implementation of all known partial-propensity SSAs. This open-source library is implemented in C++, providing both an Application Programming Interface (API) for incorporation into other software projects and a stand-alone simulator application for direct use. The software imports biochemical model specifications from Systems Biology Markup Language (SBML) files and provides tools for statistical analysis and visualization of the simulation results. Moreover, it provides a plug-in for the SBMLToolbox [25], enabling the user to change the model using a graphical user interface, and it supports the use of parallel computing resources to parallelize over simulation trajectories using the Message Passing Interface (MPI) [26]. pSSAlib adds partial-propensity methods to the family of already available SSA simulation software packages, including Cain [27], StochKit2 [28], libRoadRunner [29], and URDME [30]. This further extends the choice for users to pick the appropriate and most efficient SSA formulation for a problem at hand.

We present the software implementation of pSSAlib, validate pSSAlib, and benchmark its computational efficiency in standard test cases, comparing with the efficient NRM implementations provided by Cain [27]. Then, we use the software in order to gain insight into a new stochastic model of early-to-late endosome conversion in the endocytic pathway [31]. Besides demonstrating practical use of pSSAlib, the results show that early-to-late endosome conversion is robust to intrinsic fluctuations arising from low copy numbers of signaling molecules in the endocytic pathway. Crucially, the stochastic effects modify the non-monotonic signal-response characteristics of the deterministic counterpart of the cut-out switch and may explain the observed variability of Rab5 density at the onset of early-to-late endosome conversion in the endocytic pathway [32].

## Design and implementation

### Partial-propensity methods

As any Monte Carlo (MC) method, SSAs requires sampling a significant number of trajectories from the CME in order to acquire sufficient statistics of the system’s behavior [11]. At each time step, sampling is performed over the reaction propensities, which scales linearly with the number of reactions in the network. Partial-propensity methods reduce this computational cost by factorizing the reaction propensities. The propensity of a reaction is defined as the product of its specific probability rate and its reaction degeneracy, which counts the number of distinct ways in which reactant molecules can interact [33]. All molecules are assumed to be homogeneously distributed within the reactor. The partial propensity of a reaction is defined as the *propensity per molecule* of one of the reactants [16]. This requires that the reaction propensity has a structure that can be factorized. This is obviously the case for elementary chemical reactions, i.e., reactions involving at most two reactants, but also for other reactions where the propensity has a product structure.

The partial propensities are stored in a special data structure, called the *partial-propensity structure*. Instead of sampling over reaction propensities, partial-propensity SSAs sample over partial propensities, which can be interpreted as sampling *reaction partners* instead of reactions. Hence, instead of asking “which reaction happens next?”, partial-propensity SSAs ask: “which molecule is going to participate in the next reaction?” and then, given that: “which partner is it going to react with?” This means that instead of choosing from the set of reactions, the methods choose from the set of species, which explains why their computational cost scales with species number rather than reaction number.

### Overview of pSSAlib

We give an overview of the partial-propensity stochastic simulation algorithms library (pSSAlib), a complete and portable implementation of all partial-propensity SSAs, complemented with tools for running simulations and analyzing the results.

#### Features

pSSAlib has been designed to facilitate the process of simulating stochastic chemical reaction networks using partial-propensity methods. pSSAlib is written in C++ and is designed to be highly efficient, also supporting parallel execution on distributed-memory computer clusters. It provides a simple and unified interface to SSAs for simulating networks of elementary chemical reactions, including delayed reactions (both consuming and non-consuming) and spatiotemporal reaction-diffusion systems in box-shaped domains. In addition to elementary reactions, pSSAlib can also simulate non-elementary reactions with at most two reactant species of which one may have a stoichiometry larger than one (i.e., reactions of the type *A* + *NB* → …, *N* = 1, 2, 3, …).

The following SSAs are currently implemented in the library:

- Gillespie’s direct method (DM) [11] as a reference;
- partial-propensity direct method (PDM) [16];
- sorting partial-propensity direct method (SPDM) [16];
- partial-propensity SSA with Composition-Rejection Sampling (PSSA-CR) [17];
- delay partial-propensity direct method (dPDM) [19]
- partial-propensity stochastic reaction-diffusion method (PSRD) [20].

Apart from the classes provided by the C++ Standard Template Library (STL), pSSAlib requires other external open-source libraries: random number generators from the GNU Scientific Library (GSL) [34], Boost header-only libraries [35], and the SBML library [36] for importing model descriptions. The supported model specifications are based on SBML Level 2 Version 4 specifications. A feature comparison between pSSAlib and other popular SSA simulation packages is shown in Table 1. pSSAlib does not support the definition of Events, and spatial reaction-diffusion simulations are limited to uniform Cartesian grids using the standard NSM [13], whereas URDME supports arbitrary geometries using tetrahedral meshes. Since the diffusion part in pSSAlib is standard, we expect speedups to mainly come from the reaction part, where the use of partial-propensity methods is particularly beneficial for strongly-coupled reaction networks and for reaction-dominated dynamics.

**Table 1:** Feature comparison of pSSAlib with other exact SSA simulation packages. (SBML: supports SBML model input, pSSA: provides partial-propensity SSAs, Diffusion: provides spatiotemporal stochastic reaction-diffusion, Delays: provides chemical reactions with time delays, Events: provides event triggers).

Software package	SBML	pSSA	Diffusion	Delays	Events
pSSAlib 2.0.0	☑	☑	Cartesian NSM	☑	□
libRoadRunner 1.3.0 [29]	☑	□	□	□	☑
StochKit 2.0.13 [28]	☑	□	□	□	☑
Cain 1.10 [27]	☑	□	□	□	☑
URDME 1.3 [30]	□	□	Tetrahedral NSM	□	□

#### User interface

pSSAlib functionality can be accessed by either (i) direct calls from C++ code using the library’s API or (ii) using the pSSAlib Command Line Interface (CLI). The input can be either an SBML model from a file or defined dynamically and passed to the library using the API. Parameters, including diffusion constants, reaction rates, and time delays are defined in the SBML model as annotations. In the case of a model file, this can either be done by manual editing or through the SBMLToolbox [25] plug-in of pSSAlib.

#### Integration with the SBMLToolbox

**Fig 1:**
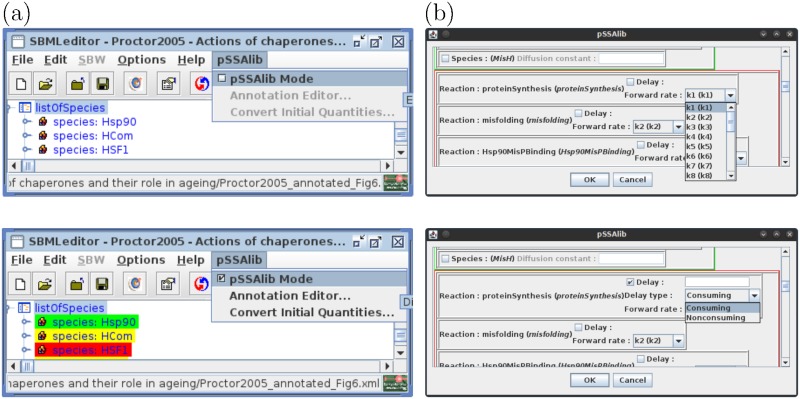
**a**. Screenshots of the new pSSAlib plug-in for the SBMLeditor, offering a graphical user interface for model editing using the SBMLToolbox [25]. **b**. Screenshots of the annotation editor for simulations using pSSAlib.

In order to facilitate manual editing of reaction rates, reaction delays, and diffusion constants, pSSAlib includes a plug-in for the SBMLToolbox [25], see Fig 1a. For ease of installation, we provide a pre-packaged distribution of the SBMLToolbox for download on the pSSAlib website. Alternatively, the plug-in can also be added to any existing installation of the SBMLToolbox by following the instructions on our website. The plug-in is activated via the respective menu item and, when active, indicates whether the model components (reactions and species) contain valid annotations that can be processed by pSSAlib. The plug-in also provides a graphical user interface for editing the respective annotations. Reaction annotations contain the reaction rates and time delays with their respective values, see Fig 1b. The plug-in uses a color code to highlight SBML nodes that can be edited, marking a node without annotation with a yellow background, red background for an invalid annotation, and green background for a valid node, see Fig 1.

### Validation

**Fig 2:**
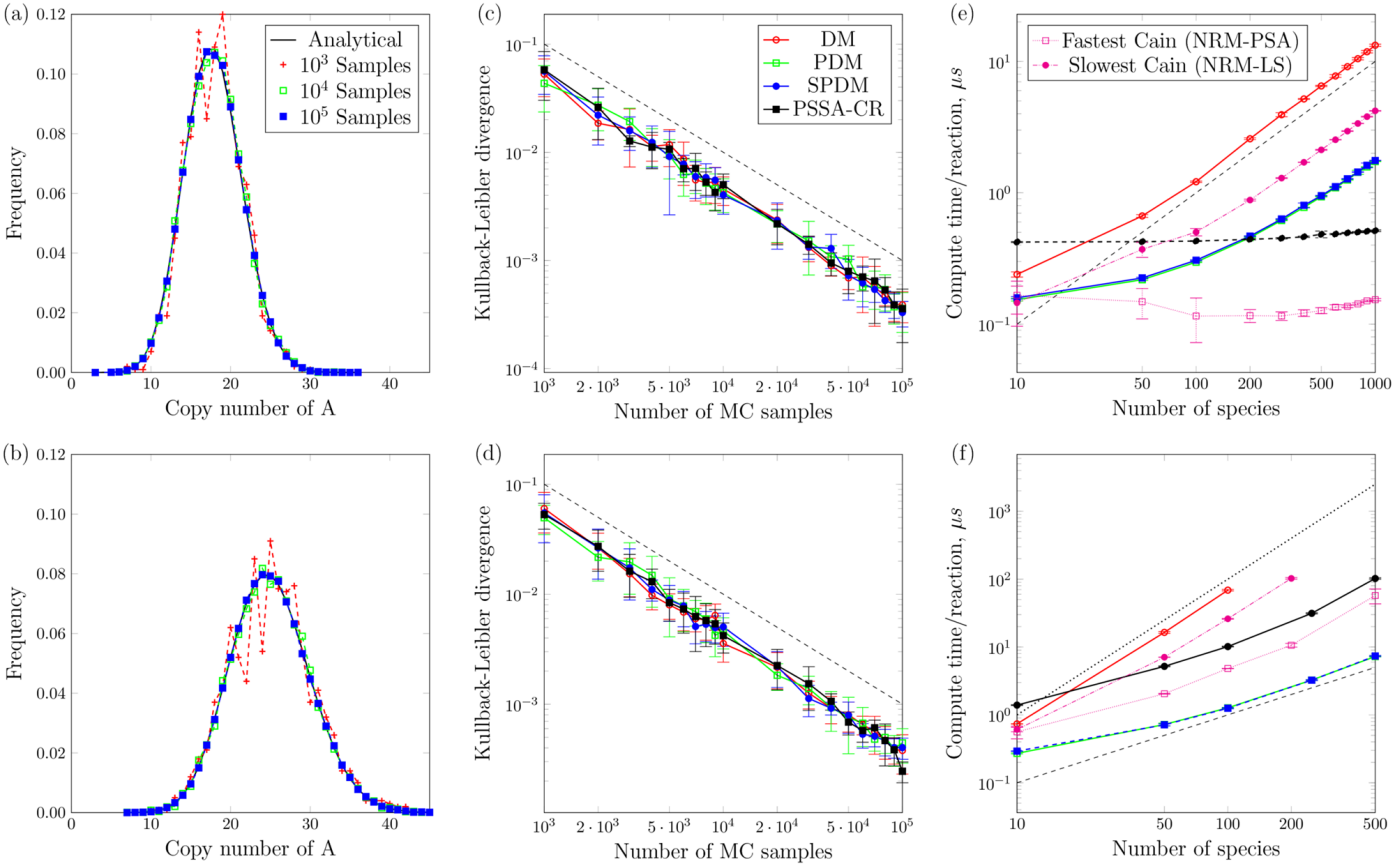
**a-d**. Results for the homo- (top row) and heteroreaction (bottom row) test cases (see Validation test cases in Sec. Supporting information): **a,b**. Simulated PDFs computed from different numbers of MC samples (symbols) using DM and plotted in comparison with the analytical PDF (solid line); **c,d**. Kullback-Leibler divergence between the analytical PDFs and the simulated ones using DM, PDM, SPDM, and PSSA-CR, computed for different numbers of samples in log—log scale. Each point represents 10 repetitions with mean and standard deviation indicated. As a guide, the black dashed line represents a slope of *S*^−1^, where *S* is the number of MC samples. **e,f**. Comparison of computational costs for various partial-propensity SSAs with respect to DM and the fastest (on this problem NRM-PSA) and slowest (on this problem NRM-LS) NRM variants implemented in Cain [27]. **e**. Results for the weakly coupled Cyclic Linear Chain model (see Benchmark test cases in Sec. Supporting information). **f**. Results for the strongly coupled Colloidal Aggregation model. In both panels, the black dashed line represents a slope of *N*^1^ (i.e., linear scaling), and the black dotted line in panel *(f)* has a slope of *N*^2^, where *N* is the number of species. Error bars in *c-f* represent standard deviations.

We use two standard reaction networks (see Validation test cases in Sec. Supporting information) to validate the correctness of the partial-propensity methods implemented in pSSAlib. For each case, different numbers of MC samples are acquired, starting from time *t* = 0 until steady state. The simulated steady-state probability distribution functions (PDFs) of species populations are shown in Fig 2a and 2b along with the corresponding analytical PDFs. To quantify similarity between empirical and analytical PDFs, we use the Kullback-Leibler divergence. Fig 2c and 2d show that the simulated PDFs converge to the analytical ones. The weak (i.e., in distribution) order of convergence of −1 (dashed black line) is expected for a MC method with a strong order of convergence of −1/2.

### Benchmarking

We benchmark the computational performance of pSSAlib on weakly coupled and strongly coupled reaction networks and compare with the highly optimized nrm implementations provided by Cain [27]. We use the cyclic linear chain model and the colloidal aggregation model as representative examples of the two classes of reaction networks (see Benchmark test cases in Sec. Supporting information). Fig 2e and 2f show the average computer runtime per simulated reaction as a function of the number of species for the pSSAlib implementations of PDM, SPDM, PSSA-CR, and DM, compared with the fastest (on this problem: next reaction method partition size adaptive (NRM-PSA)) and slowest (on this problem: next reaction method linear search (NRM-LS)) NRM implementations offered by the Cain software package [27]. The results confirm that the runtime of PDM and SPDM is linear in the number of species. As a result, they are orders of magnitude faster than traditional SSAs such as DM, whose runtime is proportional to the number of reactions. In addition, for weakly coupled networks, PSSA-CR shows constant-time computational cost. For strongly coupled networks (Fig 2f), PDM and SPDM outperform all other methods. For weakly coupled networks (Fig 2e), NRM-PSA of Cain is the fastest, while PSSA-CR shows the expected constant-time scaling.

## Results

### New insights for a biochemical switch

**Fig 3:**
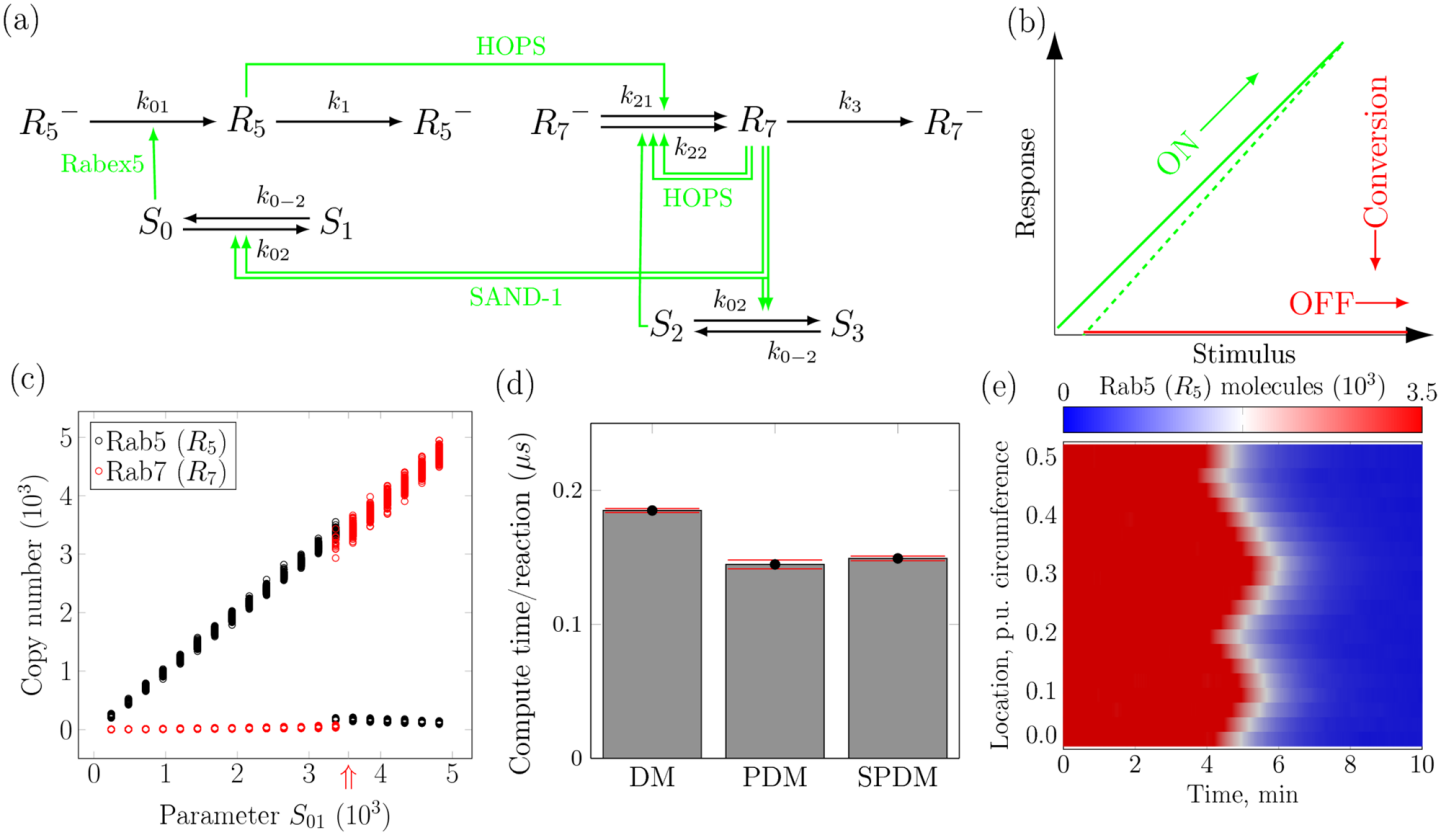
Biochemical network and self-organization underlying early-to-late endosome conversion in the endocytic pathway of eukaryotic cells. **a**. Novel reaction network diagram extending previous models of this system [32, 37]. See main text for species description; inactive GDP-bound forms are superscripted with a minus. **b**. Cut-out switch motif represented by its idealized stimulus-response diagram showing the underlying bifurcation structure. **c**. Simulated bifurcation diagram with respect to the total number of *S*_0_ and *S*_1_ molecules (parameter *S*_01_). Symbols represent final states of stochastic simulations for 100 individual samples at each parameter value, each run for 100 min of simulated time using PDM with initial conditions corresponding to the early endosome state (*R*_5_ = 1.0 mol m^−3^, *R*_7_ = 0, *S*_0_ = *S*_01_ and *S*_1_ = 0). The *S*_01_ value indicated by the red arrow is used in panels *d,e*. Other parameter values are: *k*_01_ = 1 min^−1^, *k*_02_ = 100 m^6^ mol^−2^ min^−1^, *k*_0–2_ = 10 min^−1^, *k*_1_ = 1 min^−1^, *k*_21_ = 0.1 min^−1^, *k*_22_ = 100 m^6^ mol^−2^ min^−1^, *k*_3_ = 10 min^−1^. **d**. Computer runtime per simulated reaction for DM, PDM, and SPDM with initial condition *R*_5_ = *R*_7_ = 1 mol m^−3^. **e**. Kymograph of local Rab5 molecule number within 20 subvolumes with diffusive *R*_5_ and *R*_7_ coupling, mimicking sectors on the endosomal surface. Stochastic dynamics results in spontaneous local triggering of the switch, with fronts robustly propagating the switch across space. Compartment volume was 4 × 10^−21^ m^3^ in all cases and both diffusion constants for *R*_5_ and *R*_7_ equal 10^−21^ m^2^ min^−1^.

Eukaryotic cells sort signaling molecules and nutrients through a number of distinct intracellular membrane compartments called endosomes. The decision to switch endosome types depends on the abundance of competing Rab GTPases, especially membrane-associated GTP-bound Rab5 (denoted *R*_5_) and Rab7 (denoted *R*_7_), see Fig 3a where the inactive GDP-bound forms are superscripted with a minus. Experimental evidence [31] suggests that a change in endosome type, characterized by a sharp decrease in *R*_5_ copy number, is triggered at peak abundance of *R*_5_ as the copy number of *R*_5_’s effectors increases (see Fig 3b). Theoretical investigation has revealed that such a behavior is characteristic of a cut-out switch [32, 37].

Previously, this cut-out switch has been modeled at the macroscopic level by ordinary differential equations (ODEs) using empirical species interactions [32]. Here, we propose a novel model for this switch using a reaction network that is composed of chemical reactions that obey mass-action kinetics (see Eq (1)). The corresponding species interactions are shown in Fig 3a. Here, *S*_0_ and *S*_1_ are the active and inactive forms of *R*_5_’s effector proteins, respectively. Likewise, *S*_2_ and *S*_3_ denote the active and inactive forms of *R*_7_’s effector proteins. The dynamics of *S*_2_ and *S*_3_, however, are considered identical to those of *S*_0_ and *S*_1_ in the present model and, hence, *S*_2_ is replaced by *S*_0_, and *S*_3_ by *S*_1_ in our model (see Eq (1)). By construction, the sum of copy numbers of *S*_0_ and *S*_1_ is a conserved quantity, which we denote *S*_01_. As an early endosome accumulates signaling molecules and effector proteins (*S*_0_, *S*_1_) during multiple rounds of endosome fusion and fission [38], we interpret different fixed values of *S*_01_ as different degrees of endosome progression from nascent early endosomes at low *S*_01_ to large early endosomes at high *S*_01_. The switch should be triggered by increasing the value of *S*_01_. As shown below, our mass-action-based model reproduces the experimental observations of Refs. [31, 32]. Importantly, this novel set of 7 biochemical reactions (1) allows us to use pSSAlib to study the exact stochastic system behavior for low copy numbers of molecules. Low copy numbers are expected for the small (100–500 nm diameter [39]) endosomes.

$$\begin{array}{l} S_{0}\overset{k_{01}}{\to }R_{5}+S_{0}\\ S_{0}+2R_{7}\overset{k_{02}}{\to }S_{1}+2R_{7}\\ S_{1}\overset{k_{0-2}}{\to }S_{0}\\ R_{5}\overset{k_{1}}{\to }\emptyset \\ R_{5}\overset{k_{21}}{\to }R_{7}+R_{5}\\ S_{0}+2R_{7}\overset{k_{22}}{\to }S_{0}+3R_{7}\\ R_{7}\overset{k_{3}}{\to }\emptyset \end{array}$$ (1)

We perform stochastic simulations of this model using PDM and SPDM as implemented in pSSAlib. Parameter values are given in the caption of Fig 3, and model analysis is repeated for a range of fixed parameter values *S*_01_. The simulations reveal a cut-off switch pattern in the copy numbers of *R*_5_ and *R*_7_. Specifically, as the abundance of effector proteins crosses a threshold, we observe a sharp decrease in *R*_5_ abundance accompanied by a sharp increase in *R*_7_ abundance (see Fig 3c). This observation confirms that the present model, based solely on a chemical reaction network that obeys mass-action kinetics, reconstitutes the cut-out switch that triggers transition from early to late endosomes. In addition, we observe that even for such a small chemical reaction network, both PDM and SPDM are approximately 20% faster than DM (see Fig 3d).

Until now, we have considered only temporal dynamics of the cut-out switch model by neglecting diffusion of *R*_5_ and *R*_7_. Next, we perform spatiotemporal simulations using the PSRD implementation of pSSAlib. Specifically, we consider a one-dimensional membrane section of the endosome between its two poles and account for finite diffusivity of *R*_5_ and *R*_7_. Starting from uniform spatial profiles and abundance corresponding to early endosomes, we perform stochastic simulations using PSRD. In these simulations, the abundance of effector proteins is set close to the cut-out switch threshold observed in the well-mixed simulations. Fig 3e shows the kymograph of *R*_5_ copy number obtained from the simulation. We observe that the switch in *R*_5_ abundance is triggered at multiple locations on the membrane. These locations are not deterministic, but determined by stochastic dynamics. Once the switch is triggered locally, the change in *R*_5_ abundance propagates to the rest of the membrane until the stochastic activation waves fuse (see Fig 3e). This behavior is observed over many parameter combinations and for different initial conditions (data not shown).

Through these simulations, we show for the first time that the cut-out switch model is robust against fluctuations, which are intrinsic to biological cells. In addition, we show that spatial coupling renders the concentration of Rab GTPases spatially homogeneous at steady state, despite a local trigger, thereby ensuring unique identity of individual endosomes.

## Discussion and conclusion

We have presented pSSAlib, the first complete and concise implementation of all exact partial-propensity SSAs. Partial-propensity SSAs reduce the computational cost of exact stochastic simulations of chemical reaction networks from scaling with the number of reactions to scaling at most with the smaller number of species. This is particularly beneficial for strongly coupled networks, where the number of reactions can approach the square of the number of species. In weakly coupled networks, the scaling of the computational cost of partial-propensity SSAs is identical to that of previous SSAs and is independent of system size.

pSSAlib implements and provides partial-propensity methods for strongly and weakly coupled networks, for networks involving time delays, and for spatiotemporal stochastic reaction-diffusion simulations in uniform Cartesian grids. It is implemented in C++ and can be used both stand-alone and as part of other software projects. pSSAlib is based on widely-used open-source libraries, including Boost [35], the GNU Scientific Library [34], the Message Passing Interface (MPI) [26] for parallel processing, and the SBML library [36] for portable model definition and exchange. The pSSAlib library is complemented by stand-alone software to simulate SBML models and to analyze the results. Additionally, we provide a plug-in to the SBMLToolbox for user-friendly model editing and parameter annotation (see Overview of pSSAlib in Sec. Design and implementation).

pSSAlib has previously been applied to a large biochemical reaction pathway with ATP turnover, revealing fluctuations in metabolite copy numbers on the order of 20% [23]. In the Heat-shock-response tutorial in Sec. Supporting information, we apply pSSAlib to a previously developed model of the heat shock response pathway in single cells [40]. In this manuscript, we have shown the application of pSSAlib to a novel stochastic model of endosome maturation.

We have described the architecture, functionality, and use of pSSAlib, and we have compared its functionality and computational performance with popular implementations of classical, full-propensity SSAs. Validation cases for which the exact solution of the chemical master equation is known have been used to verify correctness of the software implementation. The performance benchmarks have shown that the user-friendly implementation in pSSAlib has the beneficial scaling of partial-propensity SSAs and outperforms traditional SSAs for large or strongly coupled networks. Finally, we have applied pSSAlib and its analysis workflow to the open biological question of robust intracellular compartmentalization despite low copy numbers of the controlling molecules. To this end, we developed a new model of the endocytic pathway in eukaryotic cells and uncovered the stochastic counterpart of the cut-out switch motif underlying early-to-late endosome conversion.

Even though pSSAlib offers all partial-propensity SSAs, the choice of the most efficient method depends on problem type and size. As shown in our benchmarks, other implementations, such as the NRM variants offered by Cain [27], may be more efficient in some cases. In addition, for very small reaction networks, possibly none of the partial-propensity SSAs amortize the overhead incurred by the partial-propensity data structures and the memory look-up therein. However, pSSAlib complements the family of SSA simulation packages and provides an efficient way for existing simulation packages, like Morpheus [41] and Copasi [42], to make partial-propensity methods available to their users.

## Availability

The pSSAlib source code and pre-packaged installers are freely available from mosaic.mpi-cbg.de as open source under the GNU LGPL v3 license. The web site also contains an online manual, user tutorial, and a complete developer’s reference to the API.

## Supporting information

### Heat-shock-response tutorial

We demonstrate the application of pSSAlib to a model of age-related impairment of the heat-shock response, showing that efficient simulations enable large sample sizes and that the correspondingly accurate population averages reproduce published results [40].

Chaperones play an important role in cell physiology by assisting in proper folding of nascent proteins, refolding of misfolded ones, catalyzing protein degradation in the lysosomes, and preventing protein aggregation. However, the amount of damaged protein in a cell increases with cell age, suggesting an impairment of the heat-shock response.

An SBML model [43] of this system is publicly available from the BioModels Database [44]. After downloading this model, we added the reaction annotations using the pSSAlib plug-in for the SBMLToolbox and saved the file under a new name. The model was then ready to be processed by pSSAlib. We first reproduced the results from Fig. 2 of Ref. [40], covering tens of thousands of chaperone-protein interactions in an unstressed cell.

We let the simulation engine sample 100 trajectories using SPDM as the simulation algorithm. We chose to collect simulation output every 10 seconds of simulated time. The respective CLI call to the simulator read:

<path-to-pSSA-CLI>/simulator -m spdm -i <path-to-model-file> -n 100 --tend 10000 --dt 10 -o <output-path>

**Listing 1**. Simulator usage for 100 samples using SPDM.

We then used the pSSAlib analyzer to statistically analyze the simulation data and to plot the results. We selected the species to be included in the analysis using the -s command-line option followed by the respective species names as a comma-separated list. The two calls to the analyzer below serve to plot a single trajectory for the selected species by specifying both -n 1 and -r trajectories.

<path-to-pSSA-CLI>/analyzer -i <path-to-simulator-output>

  -s NatP, MisP, AggP, Hsp90 -n 1 -r trajectories -o <output-path> --gnuplot

<path-to-pSSA-CLI>/analyzer -i <path-to-simulator-output>

  -s ATP, ADP, ROS -n 1 -r trajectories -o <output-path> --gnuplot

**Fig 4:**
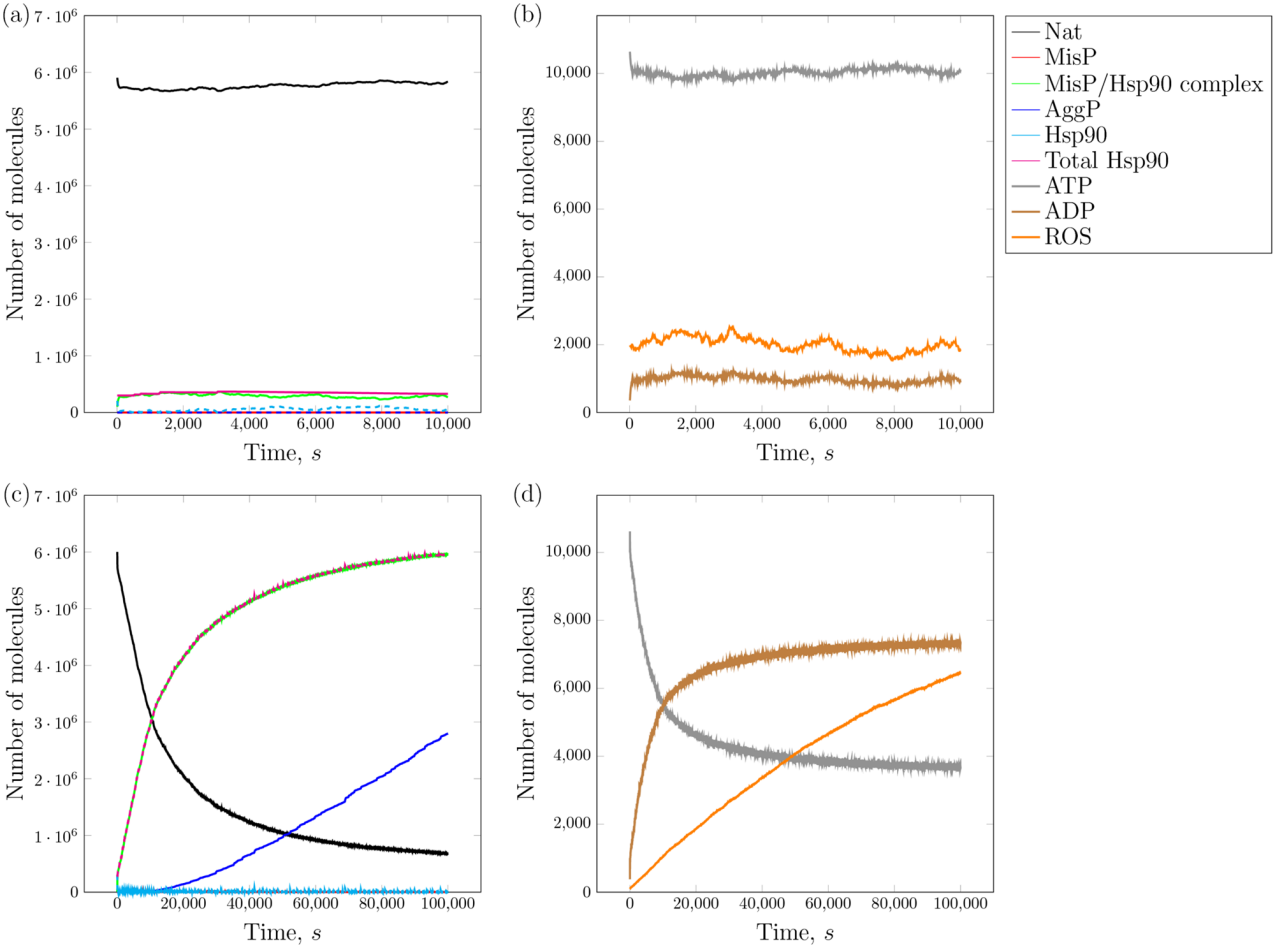
**a,b**. Heat-shock response of an unstressed cell. Visualization of the pSSAlib simulation results showing time courses for a single cell, confirming Figs. 2a and 2b from Ref. [40]. The legend of panel *b* applies to all panels. **c,d**. Heat-shock response of a stressed cell. Visualization of the pSSAlib simulation results showing time courses for a single cell, confirming Figs. 6a and 6b from Ref. [40]. The results shown here were obtained using SPDM as implemented in pSSAlib.

**Listing 2**. Analyzer usage to plot individual trajectories, as shown in Fig 4.

The --gnuplot option causes the analyzer to write gnuplot scripts for visualizing the results. These were directly visualized by the open-source plotting program gnuplot, including legends with species names as given in the model specification; see Fig 4 for an example.

The results are shown in Fig 4a and 4b and confirm Figs. 2a and 2b of Ref. [40]. Similarly, a model extension that incorporates Hsp90 misfolding in a stressed cell (see Fig 4c and 4d) reproduced Fig. 6 of Ref. [40]. Both stochastic results were thereby confirmed using a different method than in the original work.

### Validation test cases

#### Homoreaction model

The following two reactions define the homoreaction test case [45]:

$$\begin{array}{cc} \text{A}+\text{A}\overset{k_{1}}{\to }\emptyset , & \emptyset \overset{k_{2}}{\to }\text{A}. \end{array}$$ (2)

Here, *k*_1_ and *k*_2_ are macroscopic rates. In addition, we consider that these reactions happen within a finite-sized compartment of volume *V*. Using simulations, we track the copy number *n*(*t*) of molecules A. The parameter values are: *k*_1_/*V* = 0.016 s^−1^, *k*_2_*V* = 10 s^−1^. The initial copy number *n*(*t* = 0) is set to 25. The analytical steady-state population distribution is:

$$\begin{array}{c} \phi \left(n\right)=\frac{1}{\sqrt{2}I_{1}(2\sqrt{2K})}\frac{{\left(\sqrt{K}\right)}^{n}}{n!}I_{n-1}(2\sqrt{K}), \end{array}$$ (3)

where *ϕ*(*n*) is the discrete PDF for finding *n* ≥ 0 molecules of species A at steady state, $K=\frac{k_{2}V^{2}}{k_{1}}$, and *I*_*m*_(⋅) is the modified Bessel function of the first kind.

#### Heteroreaction model

The heteroreaction test case [45] is defined by the following two reactions:

$$\begin{array}{cc} \text{A}+\text{B}\overset{k_{1}}{\to }\text{B}, & \emptyset \overset{k_{2}}{\to }\text{A}. \end{array}$$ (4)

Here again *k*’s are macroscopic rates, and we consider these reactions happening within a compartment of volume *V*. Using simulations, we track the copy number *n*_a_(*t*) of species A. For this reaction network, the copy number *n*_b_ of species B remains unchanged from its initial value. The parameter values are *k*_1_/*V* = 0.04 s^−1^, *k*_2_*V* = 1 s^−1^. The initial copy numbers are *n*_a_(*t* = 0) = 25 and *n*_b_(*t*) ≡ *n*_b_(*t* = 0) = 1. The analytical steady-state population distribution for species A is:

$$\phi \left(n_{\text{a}}\right)=\frac{1}{n_{\text{a}}!}{\left(\frac{K}{n_{\text{b}}}\right)}^{n_{\text{a}}}\;\text{exp}\left(-\frac{K}{n_{\text{b}}}\right),$$ (5)

where *ϕ*(*n*_a_) is the discrete PDF for finding *n*_*a*_ molecules *A* at steady state, *n*_b_ is the constant copy number of species *B*, and $K=\frac{k_{2}V^{2}}{k_{1}}$.

### Benchmark test cases

#### Cyclic linear chain reaction network

We benchmark partial-propensity SSAs on a weakly coupled model in order to assess their limitations in cases where other SSA formulations might be more efficient. We choose the cyclic linear chain model from [16] since it is the most weakly coupled reaction network possible. For *M* reactions, it involves the minimum number of species *N* = *M* + 1, and the maximum out-degree of the dependency graph is constant at the smallest possible value of 2, since every reaction at most influences the population of its only reactant and of the only reactant of the subsequent reaction. The reactions of the cyclic linear chain model are given by:

$$\begin{array}{cc} {\text{S}}_{i}\overset{k_{i}}{\to }{\text{S}}_{i+1} & i=1,\ldots ,N-1.\\ {\text{S}}_{N}\overset{k_{N}}{\to }{\text{S}}_{1}. & \end{array}$$ (6)

where S_*i*_ is chemical species *i*, *k*_*i*_ are macroscopic reaction rates, and *N* is the total number of species in the model. For the simulations we set *k*_*i*_ = 1 and S_*i*_(*t* = 0) = 1 for all *i* = 1,…, *N*.

#### Colloidal aggregation model

We use the colloidal aggregation model from [16] as an example of a strongly coupled reaction network that can be used to model, e.g., aggregation of solvated proteins, nanobeads, or viruses. For *N* chemical species it consists of $M=\lfloor \frac{N^{2}}{2}\rfloor$ reactions. The maximum out-degree of the dependency graph is 3*N* − 7 and hence scales with system size, which is the definition of strong coupling. The reactions of the colloidal aggregation model are given by:

$$\begin{array}{rrr} {\text{S}}_{n}+{\text{S}}_{m}\overset{k_{n,m}}{\to }{\text{S}}_{n+m} & & n=1,\ldots ,\left\lfloor \frac{N}{2}\right\rfloor ;\\ & & m=n,\ldots ,N-n;\\ {\text{S}}_{p}\overset{{\bar{k}}_{p,q}}{\to }{\text{S}}_{q}+{\text{S}}_{p-q} & & p=1,\ldots ,N;\\ & & q=1,\ldots ,\left\lfloor \frac{p}{2}\right\rfloor ; \end{array}$$ (7)

where S_*i*_ is chemical species *i*, *k*_*n*,*m*_ and ${\bar{k}}_{p,q}$ are macroscopic reaction rates, and *N* is the total number of species in the model. For the simulations we set all rate constants to 1 and S_*i*_(*t* = 0) = 1 for all *i* = 1,…, *N*.
